# Temporal Study of *Salmonella enterica* Serovars Isolated from Fluff Samples from Ontario Poultry Hatcheries between 2009 and 2018

**DOI:** 10.3390/pathogens11010009

**Published:** 2021-12-22

**Authors:** Carolyn E. Murray, Csaba Varga, Rachel Ouckama, Michele T. Guerin

**Affiliations:** 1 Department of Population Medicine, Ontario Veterinary College, University of Guelph, Guelph, ON N1G 2W1, Canada; mguerin@uoguelph.ca; 2 Department of Pathobiology, College of Veterinary Medicine, University of Illinois at Urbana-Champaign, Urbana, IL 61802, USA; cvarga@illinois.edu; 3 Maple Lodge Hatcheries Ltd., Port Hope, ON L1A 3V5, Canada; rachel.ouckama@gmail.com

**Keywords:** *Salmonella enterica*, monitoring, poultry, ducks, hatchery, fluff, temporal cluster, public health, Ontario, Canada

## Abstract

The objectives of this study were to determine the prevalence, temporal trends, seasonal patterns, and temporal clustering of *Salmonella enterica* isolated from fluff samples from poultry hatcheries in Ontario between 2009 and 2018. A scan statistic was used to identify clusters of common serovars and those of human health concern. A multi-level logistic regression model was used to identify factors (poultry commodity, year, season) associated with *S. enterica* presence. The period prevalence of *S. enterica* was 7.5% in broiler hatcheries, 1.6% in layer hatcheries, 7.6% in turkey hatcheries, 29.7% in waterfowl hatcheries, and 13.8% in game-bird hatcheries. An overall increasing trend in *S. enterica* prevalence was identified in waterfowl and game-bird hatcheries, while a decreasing trend was identified in broiler and turkey hatcheries. Overall, the most common *S. enterica* serovars were Kentucky, Enteritidis, Heidelberg, and Senftenberg. *Salmonella enterica* ser. Enteritidis was the most common serovar in waterfowl hatcheries. Temporal clusters were identified for all poultry commodities. Seasonal effects varied by commodity, with the highest odds of *S. enterica* occurring in the summer and fall. Our study offers information on the prevalence and temporality of *S. enterica* serovars that might guide prevention and control programs at the hatchery level.

## 1. Introduction

Non-typhoidal *Salmonella enterica* is the most reported human enteric pathogen in Canada, with 6000 to 8000 human salmonellosis cases being reported each year [1,2,3,4]. In the province of Ontario, the annual rate of human infection in 2017 was 20.21 cases per 100,000 population, slightly higher than the national incidence rate of 19.92 cases per 100,000 [1]. Accounting for lost work, medical care, and economic losses to food companies and restaurants, the estimated economic burden of salmonellosis in Canada is CAD 1 billion annually [5].

Several serovars of *S. enterica* cause disease in humans. Of those, *S. enterica* serovars Enteritidis, Heidelberg, and Typhimurium are of greatest concern to human health, accounting for more than 50% of all detected cases of salmonellosis in Ontario and Canada in 2017 [6,7]. Most instances of human salmonellosis are caused by the consumption of contaminated poultry products (such as meat or eggs), milk, cheese, fresh produce, and direct contact with pet turtles, hedgehogs, and baby chicks [8,9,10,11]. In 2011, 63% of Canadian non-typhoidal salmonellosis cases in humans were attributed to the consumption of contaminated poultry products [8,12,13].

Both vertical and horizontal transmission are important in *S. enterica* contamination of poultry hatching eggs, and consequently, poultry hatcheries. Vertical transmission involves the in ovo transmission of specific serovars (primarily *S. enterica* serovars Enteritidis, Typhimurium, and Heidelberg) directly from a colonized breeder hen to her progeny [14,15] Horizontal transmission involves the transfer of *S. enterica* indirectly, i.e., through the environment, via transportation equipment, or by vectors, such as rodents or red mites [16,17]. The hatchery is a central point in the poultry production chain where newly hatched birds can be exposed to *S. enterica* and carry it with them to commercial farms. Therefore, control of *S. enterica* within the hatchery environment is a crucial component in controlling the colonization of commercial poultry flocks.

Our research builds on previous research conducted in Ontario to assess the temporal trends of *S. enterica* serovars in poultry hatcheries between 1998 and 2008 [18]. Using data collected as part of the Ontario Hatchery and Supply Flock Policy (OHSFP), a provincial monitoring program to detect the poultry pathogens *S. enterica* ser. Pullorum and *S. enterica* ser. Gallinarum in Ontario’s poultry hatcheries (broiler, layer, turkey, waterfowl (ducks and geese), and game bird (pheasants, partridges, and quail)), the objectives of this study were to *(i)* determine the period prevalence of *S. enterica* (all serovars) in Ontario’s hatcheries between 2009 and 2018, *(ii)* identify the most commonly isolated *S. enterica* serovars for each poultry commodity, *(iii)* examine the overall and serovar-specific long-term and seasonal trends of *S. enterica* for each poultry commodity, *(iv)* identify temporal clusters of *S. enterica* serovars, and *(v)* identify factors (poultry commodity, year, and season) associated with *S. enterica* presence.

## 2. Results

Of the 25,303 fluff samples submitted to the Animal Health Laboratory used in this study, 53.8% were from broiler hatcheries, 26.4% were from layer hatcheries, 15.8% were from turkey hatcheries, 2.8% were from waterfowl hatcheries, and 1.3% were from game-bird hatcheries (Table 1). Of all fluff samples submitted, 1678 (6.6%) were positive for *S. enterica*. The highest proportion of samples testing positive for *S. enterica* was isolated from waterfowl hatcheries (29.7%), followed by game-bird hatcheries (13.8%), turkey hatcheries (7.6%), broiler hatcheries (7.5%), and layer hatcheries (1.6%) (Table 1).

The annual prevalence of *S. enterica* between 2009 and 2018 is summarized in Table 2. The highest prevalence occurred in 2011 for broiler hatcheries, 2013 for layer hatcheries, 2015 for turkey hatcheries, 2012 for waterfowl hatcheries, and 2017 for game-bird hatcheries.

The frequency distribution of *S. enterica* serovars during the 10-year study period is presented in Table 3. Overall, 61 different serovars were isolated, although most occurred infrequently. The five most commonly isolated serovars were *S. enterica* ser. Kentucky (40.8%), *S. enterica* ser. Enteritidis (13.9%), *S. enterica* ser. Heidelberg (11.7%), *S. enterica* ser. Senftenberg (8.6%), and *S. enterica* ser. Typhimurium (4.5%). *Salmonella enterica* ser. Kentucky was isolated almost exclusively from broiler fluff samples (97.5%). *Salmonella enterica* ser. Enteritidis was isolated mainly from waterfowl and broiler samples (66.5% and 32.6%, respectively), and it was not isolated from layer or turkey samples. *Salmonella enterica* ser. Heidelberg was isolated mainly from turkey and broiler samples (50.5% and 44.9%, respectively). *Salmonella enterica* ser. Senftenberg was isolated predominantly from turkey samples (64.8%). *Salmonella enterica* ser. Typhimurium was isolated mainly from game-bird, broiler, and layer samples (34.7%, 32.0%, and 29.3%, respectively).

Of the 233 *S. enterica* ser. Enteritidis-positive samples, 83.3% were phage typed; due to newer typing methods, samples collected after September 2017 were not phage typed. The frequency distribution of *S. enterica* ser. Enteritidis phage types is summarized in Table 4. The most commonly identified phage types were 9b and 8.

### 2.1. Broiler Hatcheries

#### 2.1.1. Descriptive Statistics

A total of 13,605 fluff samples were submitted from 16 broiler hatcheries between 2009 and 2018 (Table 1). Of those, 1014 (7.5%, 95% CI: 7.0% to 7.9%) tested positive for *S. enterica*. The most commonly isolated serovars were *S. enterica* ser. Kentucky (65.6%), *S. enterica* ser. Heidelberg (8.6%), *S. enterica* ser. Enteritidis (7.5%), *S. enterica* ser. Mbandaka (2.5%), *S. enterica* ser. Livingstone (2.4%), and *S. enterica* ser. Typhimurium (2.4%) (Table 3). The majority (87.5%) of the *S. enterica* ser. Typhimurium isolates were variant Copenhagen, and 36% of the *S. enterica* ser. Mbandaka isolates were variant 14+.

#### 2.1.2. Temporal Trends

**Figure 1:**
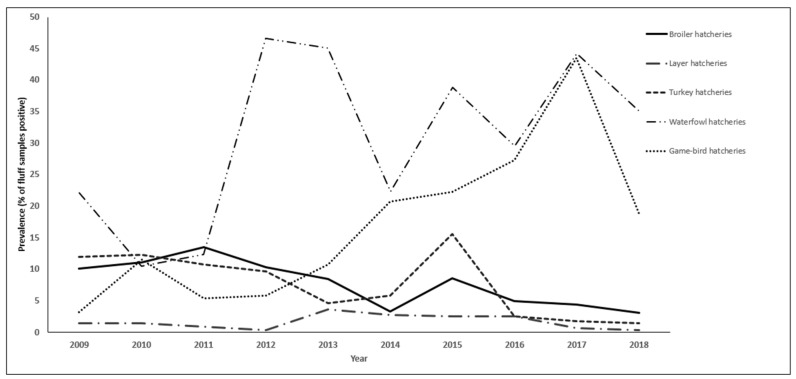
Trends in the prevalence of *Salmonella enterica* isolated from fluff samples submitted through the Ontario Hatchery and Supply Flock Policy between 2009 and 2018, by poultry commodity.

**Figure 2:**
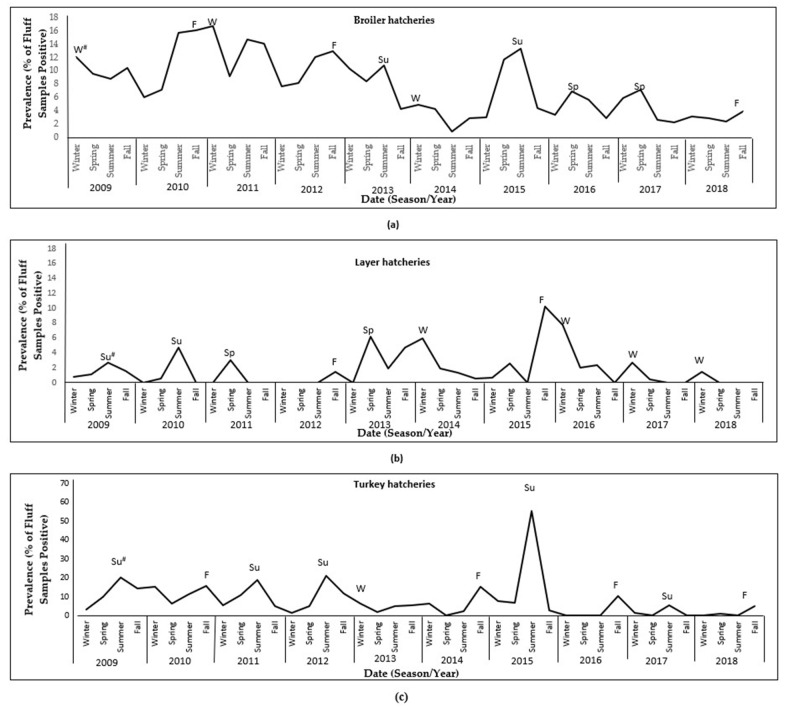

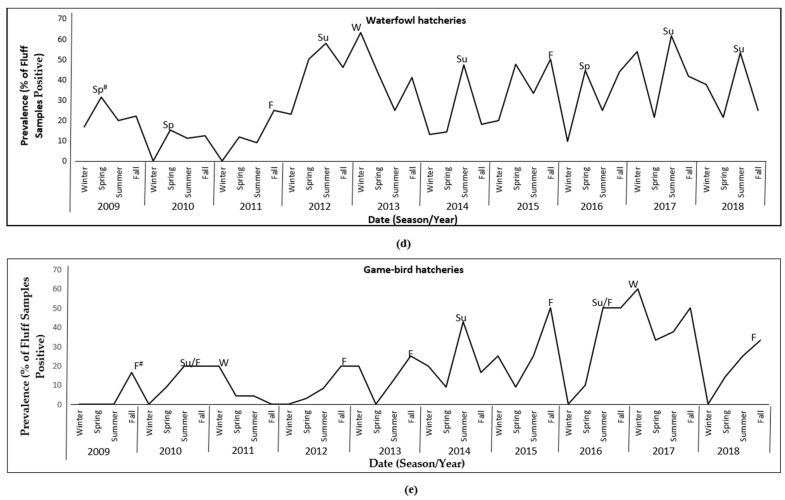
Seasonal patterns in the prevalence of *Salmonella enterica* isolated from fluff samples submitted through the Ontario Hatchery and Supply Flock Policy between 2009 and 2018, by poultry commodity. The y-axis scale may differ between graphs so that details can be observed. # Denotes the season with the highest prevalence each year: W = Winter, Sp = Spring, Su = Summer, F = Fall. (**a**): broiler hatcheries; (**b**): layer hatcheries; (**c**): turkey hatcheries; (**d**): waterfowl hatcheries; (**e**): game-bird hatcheries.

**Figure 3:**
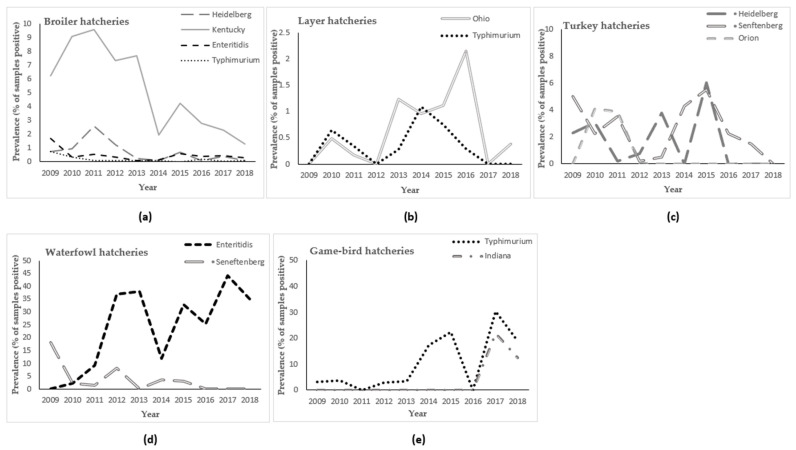
Trends in the prevalence of *Salmonella enterica* serovars isolated from fluff samples submitted through the Ontario Hatchery and Supply Flock Policy between 2009 and 2018, by poultry commodity. The y-axis scale may differ between graphs so that details can be observed. (**a**): broiler hatcheries; (**b**): layer hatcheries; (**c**): turkey hatcheries; (**d**): waterfowl hatcheries; (**e**): game-bird hatcheries.

Overall, there was a decreasing trend in the prevalence of *S. enterica* from broiler fluff samples during the study period (Figure 1) and no clear seasonal pattern was observed (Figure 2a). Trends for the most common serovars, plus *S. enterica* ser. Typhimurium, are illustrated in Figure 3a. Following a peak in 2011, there was a steep decreasing trend in the prevalence of *S. enterica* ser. Kentucky during the study period. The annual prevalence of *S. enterica* serovars Enteritidis, Heidelberg, and Typhimurium was very low (<3%) throughout the study period, and all three serovars had decreasing trends.

#### 2.1.3. Temporal Clusters

A total of 43 serovars were isolated from broiler fluff samples (Table 3). Of those, eight were included in the cluster detection analysis. Significant clusters were detected for the following *S. enterica* serovars: Enteritidis; Heidelberg; Kentucky; Livingstone; Mbandaka; Senftenberg; and Typhimurium (Table 5). Clusters ranged from short (<6 months) to long (≥6 months) duration. Three *S. enterica* serovars (Enteritidis, Heidelberg, and Kentucky) had more than one cluster and the clusters for each serovar were spread throughout the study period. All three of the *S*. *enterica* ser. Enteritidis clusters were of short duration. One of the three *S*. *enterica* ser. Heidelberg clusters was of long duration; it occurred early in the study period and included 55 isolates. Two of the *S. enterica* ser. Kentucky clusters were of long duration, the largest of which occurred during the first half of the study period over 58 months. A single, long-duration cluster of *S. enterica* ser. Mbandaka occurred during the latter half of the study period and included all but one of the *S. enterica* ser. Mbandaka isolates.

### 2.2. Layer Hatcheries

#### 2.2.1. Descriptive Statistics

A total of 6678 fluff samples were submitted from 12 layer hatcheries between 2009 and 2018 (Table 1). Of those, 105 (1.6%, 95% CI: 1.3% to 1.9%) tested positive for *S. enterica*. The most commonly isolated serovars were *S. enterica* ser. Ohio (41.9%) and *S. enterica* ser. Typhimurium (21.0%) (Table 3). The majority (93.2%) of the *S*. *enterica* ser. Ohio isolates were variant 14+, and the majority (90.5%) of the *S*. *enterica* ser. Typhimurium isolates were variant Copenhagen.

#### 2.2.2. Temporal Trends

Overall, the prevalence of *S. enterica* from layer fluff samples was consistently low (0.3% to 3.7% per year) during the study period, with the highest annual prevalence occurring from 2013 to 2016 (Figure 1). During the first half of the study period, peaks occurred mainly in the spring and summer, whereas during the latter half, peaks occurred mainly in the winter (Figure 2b). Trends for the most common serovars are illustrated in Figure 3b.

#### 2.2.3. Temporal Clusters

A total of 15 serovars were isolated from layer hatcheries (Table 3). Of those, only *S*. *enterica* serovars Ohio and Typhimurium were included in the cluster detection analysis, and significant clusters were detected for both (Table 5). The larger of the two *S. enterica* ser. Typhimurium clusters occurred from January to March 2014; it included only six isolates.

### 2.3. Turkey Hatcheries

#### 2.3.1. Descriptive Statistics

A total of 3991 fluff samples were submitted from five turkey hatcheries between 2009 and 2018 (Table 1). Of those, 305 (7.6%, 95% CI: 6.8% to 8.5%) tested positive for *S. enterica*. The most commonly isolated serovars were *S. enterica* ser. Heidelberg (32.5%), *S. enterica* ser. Senftenberg (30.8%), and *S. enterica* ser. Orion (14.4%) (Table 3). All of the *S. enterica* ser. Orion isolates were variant 15+.

#### 2.3.2. Temporal Trends

Overall, there was a decreasing trend in the prevalence of *S. enterica* from turkey fluff samples during the study period, with a peak in 2015 (Figure 1). Most peaks occurred in the summer or fall (Figure 2c). Trends for the most common serovars are illustrated in Figure 3c. There was an overall decreasing trend in the prevalence of *S. enterica* ser. Heidelberg, with no positive samples after 2015. Similarly, there was an overall decreasing trend in the prevalence of *S. enterica* ser. Senftenberg, with no positive samples in 2018.

#### 2.3.3. Temporal Clusters

A total of 20 serovars were isolated from turkey hatcheries (Table 3). Of those, only four serovars (*S. enterica* serovars Heidelberg, Newport, Orion, and Senftenberg) were included in the cluster detection analysis, and significant clusters were detected for all four (Table 5). Two serovars (*S. enterica* serovars Heidelberg and Senftenberg) had more than one cluster. All three *S. enterica* ser. Heidelberg clusters and the single *S. enterica* ser. Orion cluster were of long duration. Only one of the six *Salmonella* *enterica* ser. Senftenberg clusters was of long duration; it occurred early in the study period over 42 months and included 52 isolates.

### 2.4. Waterfowl Hatcheries

#### 2.4.1. Descriptive Statistics

A total of 704 fluff samples were submitted from eight waterfowl hatcheries between 2009 and 2018 (Table 1). Of those, 209 (29.7%, 95% CI: 26.3% to 33.1%) tested positive for *S. enterica*. The most commonly isolated serovars were *S. enterica* ser. Enteritidis (74.2%) and *S. enterica* ser. Senftenberg (13.4%) (Table 3).

#### 2.4.2. Temporal Trends

Overall, there was an increasing trend in the prevalence of *S. enterica* from waterfowl fluff samples during the study period, with peaks in 2012, 2015, and 2017 (Figure 1). Most peaks occurred in the summer or spring (Figure 2d). Trends for the most common serovars are illustrated in Figure 3d. There was an increasing trend in the prevalence of *Salmonella enterica* ser. Enteritidis and a decreasing trend in the prevalence of *Salmonella enterica* ser. Senftenberg.

#### 2.4.3. Temporal Clusters

A total of 13 serovars were isolated from waterfowl hatcheries (Table 3). Of those, only *S. enterica* serovars Enteritidis and Senftenberg were included in the cluster detection analysis, and multiple, significant, long-duration clusters were detected for both (Table 5). The *S. enterica* ser. Enteritidis clusters ranged from 24 to 48 months in duration.

### 2.5. Game-Bird Hatcheries

#### 2.5.1. Descriptive Statistics

A total of 325 fluff samples were submitted from eight game-bird hatcheries between 2009 and 2018 (Table 1). Of those, 45 (13.8%, 95% CI: 10.1% to 17.6%) tested positive for *S. enterica*. The most commonly isolated serovars were *S. enterica* ser. Typhimurium (57.8%) and *S. enterica* ser. Indiana (15.6%) (Table 3). The majority (96.2%) of the *S. enterica* ser. Typhimurium isolates were variant Copenhagen.

#### 2.5.2. Temporal Trends

Overall, there was an increasing trend in the prevalence of *S. enterica* from game-bird fluff samples during the study period, peaking at 43.5% in 2017 (Figure 1). Most peaks occurred in the fall or summer (Figure 2e). Trends for the most common serovars are illustrated in Figure 3e. There was an increasing trend in the prevalence of *S. enterica* ser. Typhimurium, peaking in 2017. *Salmonella enterica* ser. Indiana was not detected among game-bird hatcheries before 2017.

#### 2.5.3. Temporal Clusters

A total of nine serovars were isolated from game-bird hatcheries (Table 3). Of those, only *S*. *enterica* ser. Typhimurium was included in the cluster detection analysis, and a significant, long-duration cluster was detected (Table 5). The cluster occurred over 56 months (May 2014 to December 2018) and included 21 isolates. Of those, 20 were from the same hatcher.

### 2.6. Regression Analysis

Based on a Spearman’s rho value of less than |0.8|, there was no evidence of high correlation among the predictor variables. In the fixed-effects logistic model, all three predictor variables were significant (*p* ≤ 0.05), and there was significant interaction between year and poultry commodity, season and poultry commodity, and year and season with *S. enterica* status. The best-fitting model, based on the Akaike information criterion (AIC) (Table 6), included random intercepts for both hatchery and date of sample collection, as well as the interaction terms between year and commodity and season and commodity (Table 7). The interaction term between year and season was removed based on the AIC.

The effect of commodity on the odds of a sample testing positive for *S. enterica* varied by year. Compared to fluff samples submitted from broiler hatcheries in 2009, the odds of *S. enterica* were higher in samples submitted from layer hatcheries in 2014, in samples submitted from waterfowl hatcheries between 2012 and 2018, and in samples submitted from game-bird hatcheries between 2014 and 2018. The effect of commodity on the odds of a sample testing positive for *S. enterica* also varied by season. Compared to fluff samples submitted from broiler hatcheries in winter, the odds of *S. enterica* were higher in samples submitted from turkey hatcheries in summer and fall, and in samples submitted from waterfowl hatcheries in summer. After controlling for fixed effects, the proportion of variation explained at the hatchery level was 49% (σ^2^_hatchery_ = 5.038, 95% CI: 2.750 to 9.229), and the variation explained at the visit level was 19% (σ^2^_visit_ = 1.942, 95% CI: 1.600 to 2.359).

From the final model, the predicted probability of *S. enterica* for each year and season was calculated for each poultry commodity and is illustrated in Figure S1.

## 3. Discussion

This study analyzed hatchery fluff data collected from 2009 to 2018 for the OHSFP monitoring program, to determine the period prevalence, temporal trends, and seasonal patterns of *S. enterica* for all poultry commodities, as well as to identify temporal clusters of *S. enterica* serovars of interest. Relative to the previous 11-year period (1998 to 2008) [18], the period prevalence of *S. enterica* from fluff samples was lower for broiler hatcheries (7.5% vs. 8.7% for the previous period), layer hatcheries (1.6% vs. 3.1% for the previous period), and turkey hatcheries (7.6% vs. 13.2% for the previous period), and higher for other hatcheries (29.7% and 13.8% for waterfowl and game-bird hatcheries, respectively, vs. 11.9% for ducks, geese, quail, partridges, and pheasants combined for the previous period). Descriptive analysis revealed that the temporal trends of *S. enterica* prevalence between 2009 and 2018 varied among the different commodities. A decreasing trend in *S. enterica* prevalence was observed in broiler and turkey hatcheries, while the prevalence remained consistently low in layer hatcheries. Conversely, an increasing trend in *S. enterica* prevalence was observed in waterfowl and game-bird hatcheries. The decrease in broiler-hatchery serovar prevalence coincides with the introduction of province-wide vaccination of all domestic broiler breeders against *S. enterica* serovars Enteritidis, Heidelberg, Infantis, Kentucky, and Typhimurium, beginning mid-way through the study period. The results from the multi-level logistic regression model revealed that the effect of poultry commodity on the *S. enterica* prevalence depended on both year and season, and that there was significant variation in *S. enterica* prevalence among hatcheries.

Retrospective scan statistics identified temporal clusters of *S. enterica* serovars for all poultry commodities. Long-duration (≥6 months) clusters were identified during the latter half of the study period for several commodities: *S. enterica* ser. Mbandaka in broiler hatcheries; *S. enterica* ser. Enteritidis in waterfowl hatcheries; and *S. enterica* ser. Typhimurium in game-bird hatcheries. These findings indicate that serovars of public health concern have recently become endemic in the hatcheries of several poultry commodities. The remaining clusters were identified throughout the study period and were of both short (<6 months) and long duration. Long-duration clusters likely indicate a continuous common source, farm-to-farm transmission, or secondary infections, whereas short-duration clusters likely indicate a point-source of contamination [19].

A key finding in our study was that *S. enterica* ser. Enteritidis was not identified in layer or turkey hatcheries. For layer hatcheries, it is likely that active prevention (e.g., vaccination of breeder birds) and control measures (e.g., depopulation of infected breeder flocks) taken to eliminate this serovar contributed to this finding. Further, this might be due to a reduced number of hatching eggs imported from the United States compared to the other poultry commodities. Similarly, for turkey hatcheries, strict controls of hatching egg imports from the United States likely limited the number of *S. enterica* ser. Enteritidis-contaminated eggs reaching Ontario hatcheries. These findings are consistent with a previous study in Ontario between 1998 and 2008 that did not identify *S. enterica* ser. Enteritidis in these hatchery types [18]. *Salmonella enterica* ser. Enteritidis might not be as competent at disseminating within or colonizing populations of turkeys, as our findings are comparable to surveillance data from the United States which showed that *S. enterica* ser. Enteritidis was not significantly detected within turkey flocks during this period [20,21,22,23].

However, *S. enterica* ser. Enteritidis was the third most common serovar in broiler hatcheries. Three relatively small clusters (11 to 17 isolates per cluster) were identified, each of short duration. Due to the near eradication of this serovar in domestic broiler breeder flocks from extensive testing and depopulation of infected flocks, and the introduction of the Ontario broiler-breeder vaccination program described above, the primary source of this serovar in Ontario broiler hatcheries is contaminated hatching eggs imported from the United States [24]. Once a positive sample is detected, further imports from the infected U.S. flock are halted, preventing the sustained horizontal transmission needed for a long-duration cluster. The most common phage types were PTs 8 and 13, followed by 13a. Interestingly, these were the most common phage types among human *S. enterica* ser. Enteritidis cases in Ontario from 2008 to 2009 [25]. Due to the change to whole-genome sequencing in August 2017, no phage-type data were available for broiler fluff samples for the end of the study period. However, before the conversion to whole-genome sequencing, the National Enteric Surveillance Program in Canada identified PTs 8, 13, and 13a as the most common phage types detected from human isolates in Ontario [26]. The similarities in the *S. enterica* ser. Enteritidis phage types between broiler hatcheries and human cases suggest that public health could be positively impacted by control measures at the broiler-hatchery level.

In contrast, *S. enterica* ser. Enteritidis was the most common serovar in waterfowl hatcheries, accounting for nearly three quarters of the positive samples in this commodity. An overall increasing trend in *S. enterica* prevalence was observed in waterfowl hatcheries, which was driven by the increasing trend in *S enterica* ser. Enteritidis prevalence. Three long-duration clusters (2 to 4 years per cluster) of *S. enterica* ser. Enteritidis were identified, suggesting that a continual source of the bacteria, such as an endemic environmental source or vertical transmission from colonized breeder flocks, might have caused these clusters. However, submission of environmental samples is optional for waterfowl and game-bird breeder flocks, resulting in surveillance data for these poultry commodities being limited and inconsistent, thereby restricting the inferences that can be drawn about the sources and transmission of *S. enterica* serovars in these hatcheries. Phage type 9b was the predominant phage type among *S. enterica* ser. Enteritidis isolates from waterfowl fluff samples. This is consistent with a previous Ontario study, which identified PT 9b as the most common phage type among “other” breeder hatcheries (ducks, geese, pheasants, partridges, quail) from 1998 to 2008 [18]. Phage type 9b was sporadically identified as the agent in human salmonellosis cases nationally during the study period [26]. The disparity between the prevalence of PT 9b in waterfowl-hatchery samples and the prevalence within human samples might reflect the reduced consumption of waterfowl compared to broiler meat, or that PT 9b might not be as competent as other phage types at infecting humans.

In our study, *S. enterica* ser. Heidelberg was another prominent serovar in both broiler and turkey hatcheries. *Salmonella enterica* ser. Heidelberg was the second most common serovar identified from broiler hatcheries. However, the annual prevalence in broiler hatcheries was very low (<3%) and decreased throughout the study period, whereas in turkey hatcheries, it was the most common serovar isolated. This is consistent with a previous Ontario study, which identified it as the most common serovar isolated from turkey-hatchery fluff samples between 1998 and 2008 [18]. During the study period, a decreasing trend in the prevalence of *S. enterica* ser. Heidelberg was observed in turkey hatcheries, with no positive samples of the serovar being identified from this poultry commodity from 2016 to the end of the study. This is mirrored among environmental samples collected from turkey breeder farms during the same period (unpublished data) [27] and is likely a result of increasing efforts to control this serovar among turkey breeders and hatcheries. As discussed by Sivaramalingam et al., the temporal similarities between the hatchery and breeder flock level suggest that control measures implemented at the breeder flock level for all poultry commodities are likely to contribute to a decrease in *S. enterica* prevalence at lower levels of the poultry production chain [18].

*Salmonella enterica* ser. Kentucky was almost exclusively identified from broiler-hatchery fluff samples and was the most common serovar for this commodity. Further, it was the most common serovar among environmental samples collected from broiler breeder flocks during the same period (unpublished data) [27]. This is consistent with previous Ontario studies, which identified it as the most prevalent serovar among broiler hatcheries and broiler breeder flocks between 1998 and 2008 [18,28]. The serovar showed an overall decreasing trend, especially between 2013 and 2014, excluding an increase from 6.2% to 9.6% early in the study period, between 2009 and 2011. Similarly, a decreasing trend was observed in broiler flocks in Ontario from surveillance data between 2013 and 2018 and in the United States between 2009 and 2016, excluding an increase from 2012 to 2014 [20,21,22,23,29,30]. In these studies, *S. enterica* ser. Kentucky was consistently ranked as the top, or one of the top, most common serovars in chickens (a majority of which were broilers).

*Salmonella enterica* ser. Senftenberg was the second most common serovar in turkey and waterfowl hatcheries. Among the total samples identified as positive for *S. enterica* ser. Senftenberg, samples collected from turkey hatcheries accounted for more than 60%. *Salmonella enterica* ser. Senftenberg was identified as the most common serovar among turkey hatcheries in Ontario from 1998 to 2008 [18]. A decreasing trend with multiple peaks was observed among turkey fluff samples, with no samples being identified as positive for *S. enterica* ser. Senftenberg in 2018. The sole, long-duration cluster was observed early in the study period (2009–2012), with only short-duration clusters being detected for the remainder of the study period. As discussed by Guerin et al., this could indicate a shift from farm-to-farm or wide-spread, common-source transmission, to point-source infections [19]. Among waterfowl fluff samples, a decreasing trend was observed, with zero isolates being identified from 2016 to the end of the study period.

Unlike other serovars, *S. enterica* ser. Typhimurium was not common in broiler or turkey hatcheries, and it was not identified in waterfowl hatcheries. However, it was the most common serovar in game-bird hatcheries and the second most common serovar in layer hatcheries. *Salmonella enterica* ser. Typhimurium accounted for nearly 60% of all *S. enterica*-positive game-bird samples, with 96.2% of those being classified as variant Copenhagen. This is consistent with previous North American studies on quail, which identified *S. enterica* ser. Typhimurium variant Copenhagen as the most prevalent serovar [31]. An increasing trend in the prevalence of this serovar was observed, and a single, long-duration cluster lasting 56 months (from May 2014 to December 2018) was detected. As nearly all samples (95.2%) belonging to this cluster were from a single game-bird hatchery, this finding likely indicates pervasive contamination of the facility. Conversely, from layer fluff samples, only short-duration clusters were identified. This indicates that the sources and transmission of *S. enterica* ser. Typhimurium might differ between poultry commodities. *Salmonella enterica* ser. Typhimurium is particularly pathogenic to humans and ranks consistently as the second most frequent serovar causing salmonellosis among humans, after *S. enterica* ser. Enteritidis. Therefore, early identification of clusters and transmission patterns among poultry commodities would be useful in preventing outbreaks among both poultry and humans.

The trends observed in *S. enterica* prevalence varied by poultry commodity. The increasing trend in *S. enterica* prevalence in waterfowl hatcheries was driven by *S. enterica* ser. Enteritidis, while the increasing trend in game-bird hatcheries was mainly due to increasing trends in *S. enterica* serovars Typhimurium and Indiana. These serovars can be transmitted vertically and horizontally to progeny [13,14]. The hatcheries might have become initially contaminated via eggs from infected breeder flocks. Some *S. enterica* serovars can remain on surfaces for long periods within environments after cleaning and disinfection, even in the absence of an infected flock, and once established in the hatchery, the bacteria can contaminate subsequent batches of eggs [16,32,33]. The increasing trends observed might additionally be caused by poor biosecurity or sanitation at the breeder flock or hatchery levels. Conversely, the decreasing trend in broiler hatcheries was predominantly driven by a reduction in samples testing positive for *S. enterica* ser. Kentucky, and the decreasing trend in turkey hatcheries was driven by trends of the two most common serovars: *S. enterica* serovars Heidelberg and Senftenberg. These serovars can be spread by horizontal transmission and their decrease could reflect efforts focused on improving biosecurity, sanitation, and reducing prevalence at the breeder flock level.

The influence of one or a few serovars on the overall trends of *S. enterica* among poultry commodities indicates that the population dynamics of *S. enterica* in poultry can be very serovar-specific. Serovars can become prevalent and then decrease in reservoir populations within variable periods. As discussed by Sivaramalingam et al., these changes in prevalence are usually unknown, although they might be caused by microbial adaptation, resulting in changes in transmissibility or survivability, competition among serovars, changes in ecological niches, serovar-specific prevention and control strategies, including testing and depopulation, changes in immunity of poultry populations caused by vaccination or infection, or changes in management factors [18]. Further, the logistic regression identified significant interactions between poultry commodity and year and poultry commodity and season on *S. enterica* presence. Knowledge of how these factors influence *S. enterica* prevalence within reservoir populations would help to guide control programs, and future studies should work to better understand the mechanics behind these types of *S. enterica* serovar trends.

Finally, a multi-level analysis allowed for the estimation of variance at different hierarchical levels. The moderate percentage of variation at the hatchery level suggests that further investigation or interventions at this level are warranted. The variation in our study at the hatchery level might be influenced by several factors: biosecurity measures and management practices adopted by the hatchery or the breeder flocks supplying the hatchery; the hatchery’s supplier of hatching eggs and chicks coming from the United States; and the location of the hatchery. However, large confidence intervals were calculated for the interaction between game-bird hatcheries and year, caused by a limited number of samples collected from these facilities. Future studies should be conducted to better understand *S. enterica* trends in this commodity and in waterfowl hatcheries.

Our data were collected at the population level and interpretations are representative of Ontario’s broiler chicken, layer chicken, and turkey hatcheries. They are likely representative of waterfowl and game-bird hatcheries of this size. Additional research should also be conducted to better understand the serovar-specific dynamics of *S. enterica* within poultry hatcheries, specifically, the underlying mechanics causing changes in serovar dominance over time. Further, there is a need to develop improved techniques to use cluster information to identify sources of *S. enterica* within the hatchery environment and to better understand the reasons for temporal clustering. Research should be conducted to identify the factors that play a role in the increased prevalence of *S. enterica* observed within hatcheries during the summer and fall seasons. At the farm level, research is needed to determine which management practices, biosecurity protocols, and cleaning and disinfection routines are associated with decreased *S. enterica* prevalence. Finally, from a public health perspective, work is required to identify whether there is a link between temporal clusters of *S. enterica*, detected at any level of poultry production, and human cases of salmonellosis.

## 4. Materials and Methods

### 4.1. Data Source

Monitoring data from Ontario hatcheries registered under the OHSFP between 2009 and 2018 were obtained from the Animal Health Laboratory, Guelph, Ontario. This program stipulates that 0.5 g of fluff be collected and submitted to the Animal Health Laboratory every six weeks from every hatcher with a setting capacity of at least 1000 eggs per day. Therefore, each submitted sample represented fluff material collected from an individual hatcher from a specific hatchery on a specific date.

The Animal Health Laboratory is an American Association of Veterinary Laboratory Diagnosticians-accredited diagnostic facility that operates as the provincial animal health lab for Ontario. A total of 0.5 g of fluff was immersed in 100 mL of buffered peptone water and incubated for 24 h at 35 °C. One hundred microliters of the suspension was then inoculated on three equally spaced spots on semi-solid Rappaport–Vassiliadis (MSRV) agar and incubated for 24 and 48 h at 42 °C in order to detect motile salmonellae. After 24 and 48 h (if negative at 24 h) incubation, MSRV plates with an opaque turbid area around the inoculation spots were used to stab a 1 μL loop at the edge of the turbid area and this material was transferred to selective brilliant green sulfa-novobiocin (BGS-N) and xylose lysine tergitol-4 (XLT-4) agar plates. These plates were then incubated at 35 °C and examined at 24 and 48 h. Presumptive *Salmonella* spp. colonies (i.e., pink to red, with or without a black center) were confirmed by using matrix-assisted laser desorption ionization time-of-flight mass spectrometry (MALDI-TOF) (Bruker Ltd., Billerica, MA, USA) using one suspicious colony per plate if colonies were morphologically identical, or multiple colonies if different morphologies were present. Serotyping of *Salmonella* isolates was conducted at the OIE (World Organisation for Animal Health) *Salmonella* reference laboratory at the National Microbiology Laboratory in Guelph, using the Kauffmann–White–Le Minor classification scheme. Phage typing of isolates was discontinued by the *Salmonella* reference laboratory by August 2017.

The dataset was provided to the research team as Microsoft Excel 97-2003 Worksheets (2 to 3 years of data per spreadsheet). The following information was extracted for the fluff samples: submission identification (ID); sample ID; the date on which the sample was received by the lab; hatchery ID; hatchery location (city); commodity description (i.e., type of poultry); culture results; and a reference code for each isolated serovar. To minimize the occurrence of improperly reported commodities, hatcheries were cross-referenced with the Canadian Food Inspection Agency’s (CFIA’s) List of Health Monitored Hatcheries [34] and corrected if necessary. For example, if the hatchery name and city in the dataset corresponded to a hatchery included on the CFIA’s list, yet the commodity description was missing or ambiguous (e.g., “chicken” instead of “chicken-broiler”), the commodity description in the dataset was changed to match the CFIA’s list.

### 4.2. Descriptive Analysis

Descriptive analyses were performed using both Microsoft Office Excel 2016 (Microsoft Corporation, Redmond, WA, USA) and Stata IC version 16 (STATA Corporation, College Station, TX, USA). Commodities were grouped as broilers, layers, turkeys, waterfowl (ducks, geese), and game birds (pheasants, partridges, quail). Culture results that were positive for other bacteria, such as *Pseudomonas* spp., or yielded no *S. enterica* growth, were considered to be negative for *S. enterica*. The number of hatcheries, total number of fluff samples submitted, number of *S. enterica*-positive fluff samples identified, and commodity-specific sample prevalence for the 10 years from 2009 to 2018 were summarized. Exact binomial 95% confidence intervals for the prevalence estimates were calculated. The proportion of *S. enterica*-positive fluff samples and the frequencies of serovars isolated from each poultry commodity for the same period were tabulated.

Long-term trends of *S. enterica* prevalence among the different poultry commodities were illustrated after estimating the annual prevalence for the 2009 to 2018 period. Additionally, seasonal prevalence patterns were visualized for each poultry commodity. For this purpose, and in keeping with previous Canadian studies, winter was defined as January to March, spring as April to June, summer as July to September, and fall as October to December. Seasonal prevalence was estimated by dividing the total number of *S. enterica*-positives from all hatcheries for each season by the total number of samples that were submitted during the same season and multiplying by 100.

### 4.3. Temporal Cluster Detection

A retrospective temporal scan statistic using SaTScan software version 9.6 [35] was applied to identify periods with a higher-than-expected number of *S. enterica*-positive fluff samples. For each poultry commodity, cluster detection was conducted for all serovars with a frequency of at least 10 isolates during the study period. A case was defined as an *S. enterica*-positive sample of a specific serovar within a specific poultry commodity, during a specific period. A non-case was defined as an *S. enterica*-positive sample other than the specific serovar, or an *S. enterica*-negative sample, within the same commodity and period. For instance, an *S. enterica*-negative sample, and a sample positive for *S. enterica* ser. Heidelberg, would both be considered negative for *S. enterica* ser. Typhimurium. The smallest time unit was represented by the month and year of *S. enterica* testing.

A Bernoulli model [36] was used to estimate a relative risk and a log-likelihood ratio for the temporal scan. This model was selected because the data consisted of two possible outcomes, cases and non-cases, during the study period. The model compares the proportion of cases of a specific serovar within the time window to the proportion of cases of that same serovar outside the time window. The temporal scan statistic’s 50% scanning window (default setting) was chosen to examine every possible period within the study period.

A likelihood-ratio test statistic was used to assess the significance of each temporal cluster; the statistic reflects the difference between the observed number of *S. enterica* cases and what would be expected under the null hypothesis of no temporal trend. A simulated *p*-value of ≤ 0.05, calculated through a Monte Carlo simulation using 999 replications, signified that the cluster was significant, and the null hypothesis was rejected. The iterative scan option was used to identify a primary cluster (highest likelihood ratio) and all possible significant secondary clusters.

### 4.4. Logistic Regression Analysis

Using the *melogit* command in Stata IC version 16, a multi-level logistic regression analysis was conducted to test the association between the *S. enterica* test result obtained from a fluff sample (positive/negative for *S. enterica*) and the risk factors of poultry commodity, year, and season (see below). Random intercepts were included for hatchery and date of sample collection to account for clustering among hatcheries, and among the hatchers on the day of the visit, respectively. All variables used in the analysis were categorical; broiler hatcheries, the year 2009, and winter were selected as the referent categories for poultry commodity, year, and season, respectively.

Univariable analysis to screen variables was not conducted because all three predictors were *a priori* variables of interest. Using a backward elimination approach, the overall statistical significance of each predictor variable was assessed using a likelihood-ratio test, with *p* ≤ 0.05 indicating significance. Next, all two-way interactions between significant main effects were added to the model separately and assessed for significance using a likelihood-ratio test. Then, all combinations of two-way interactions (from those that were significant) were added to the model two at a time. A model with three interaction terms was not considered because there were too few waterfowl and game-bird submissions for meaningful analysis. The main-effects model, and models with one or two interactions, were compared using the AIC; the best-fitting model was selected based on the lowest AIC score.

The best linear unbiased predictors (BLUPS) for the hatchery-level residuals were used to evaluate the assumptions of normality and homoscedasticity; a Q–Q plot was used to assess normality and a plot of standardized residuals vs. fitted values was used to assess homoscedasticity. Intra-class correlation coefficients (ICCs) were estimated using the latent-variable technique to determine the percentage of variation at each level of clustering. The predicted probabilities of *S. enterica*-positive status were attained from the best-fitting model and visualized across poultry commodity, year, and season.

## Figures and Tables

**Table 1:** Overall period prevalence of *Salmonella enterica* isolated from fluff samples submitted through the Ontario Hatchery and Supply Flock Policy between 2009 and 2018, by poultry commodity (*n* = 25,303 samples).

Poultry Commodity	Number of Hatcheries	Number of Samples Submitted	Percentage of Total Samples Submitted	Number of *S. enterica*-Positive Isolates	Commodity-Specific Sample Prevalence (%)
Broiler hatcheries	16	13,605	53.8	1014	7.5 (7.0–7.9) ^1^
Layer hatcheries	12	6678	26.4	105	1.6 (1.3–1.9)
Turkey hatcheries	5	3991	15.8	305	7.6 (6.8–8.5)
Waterfowl hatcheries	8	704	2.8	209	29.7 (26.3–33.1)
Game-bird hatcheries	8	325	1.3	45	13.8 (10.1–17.6)

^1^ Exact binomial 95% confidence interval.

**Table 2:** Annual prevalence of *Salmonella enterica* isolated from fluff samples submitted through the Ontario Hatchery and Supply Flock Policy between 2009 and 2018, by poultry commodity (*n* = 25,303 samples).

	Number of Samples Submitted (% Positive)					
Year	Broiler Hatcheries	Layer Hatcheries	Turkey Hatcheries	Waterfowl Hatcheries	Game-Bird Hatcheries	Annual Total
2009	1482 (10.1)	599 (1.5)	260 (11.9)	77 (22.1)	31 (3.2)	2449 (8.5)
2010	1256 (11.1)	622 (1.4)	488 (12.3)	86 (10.5)	26 (11.5)	2478 (8.9)
2011	1115 (**13.5**)	574 (0.9)	565 (10.8)	65 (12.3)	55 (5.5)	2374 (**9.6**)
2012	1200 (10.3)	601 (0.3)	592 (9.6)	73 (**46.6**)	68 (5.9)	2534 (8.7)
2013	1148 (8.4)	732 (**3.7**)	581 (4.6)	71 (45.1)	28 (10.7)	2560 (7.1)
2014	1283 (3.3)	732 (2.7)	326 (5.8)	85 (22.3)	29 (20.7)	2455 (4.2)
2015	1157 (8.6)	538 (2.6)	199 (**15.6**)	67 (38.8)	27 (22.2)	1988 (8.8)
2016	1486 (4.9)	695 (2.6)	310 (2.6)	71 (29.6)	22 (27.3)	2584 (5.8)
2017	1837 (4.4)	800 (0.8)	341 (1.8)	52 (44.2)	23 (**43.5**)	3053 (4.1)
2018	1641 (3.1)	785 (0.4)	329 (1.5)	57 (35.1)	16 (18.8)	2828 (2.0)
**Total:**	**13,605 (7.5)**	**6678 (1.6)**	**3991 (7.6)**	**704 (29.7)**	**325 (13.8)**	**25,303 (6.6)**

Within a column, the year with the highest prevalence is shown in bold.

**Table 3:** Frequency of *Salmonella enterica* serovars isolated from fluff samples submitted through the Ontario Hatchery and Supply Flock Policy between 2009 and 2018, by poultry commodity (*n* = 25,303 samples).

Serovar	Group	Broiler Hatcheries	Layer Hatcheries	Turkey Hatcheries	Waterfowl Hatcheries	Game-Bird Hatcheries	Total
Agona	B	0	0	7	0	0	7
Albany	C2	1	0	3	0	0	4
Anatum	E1	5	0	0	0	0	5
Arizona		1	0	0	0	0	1
Braenderup	C1	5	1	0	0	2	8
Bredeney	B	0	0	3	0	0	3
Cerro	C1	1	0	0	0	0	1
Enteritidis	D	**76**	0	0	**155**	2	**233**
Give	E1	2	0	8	0	0	10
Hadar	C2	1	0	1	9	0	11
Hartford	C1	0	0	0	0	1	1
Heidelberg	B	**88**	8	**99**	1	0	**196**
I:10:-:1 5	E1	0	0	1	0	0	1
I:10:Eh:- 10:Eh:-	E1	0	0	2	0	0	2
I:10:I,Z13:-	E1	0	0	0	0	1	1
I:4 12:I:- 4:I:-	B	1	0	0	0	0	1
I:4,12:d:-	B	1	0	0	0	0	1
I:4,12:I:-	B	2	5	0	0	0	7
I:4,5,12:-:-	B	1	0	0	0	0	1
I:4,5,12:-:1,2	B	1	0	0	0	0	1
I:4,5,12:I:-	B	7	1	0	0	0	8
I:6 7:-:1 5 6 7:-:5	C1	1	0	0	0	0	1
I:6,7,14:B:-	C1	0	1	0	0	0	1
I:6,7:K:-	C1	1	0	0	0	0	1
I:8 20:-:Z6 8 20:-:Z6	C2	1	0	0	0	0	1
I:8 20:I:- 8 20:I:-	C2	2	0	0	0	0	2
I:8,20:-:Z6	C2	4	5	0	0	0	9
I:Rough-O:-:-	C2	2	0	0	1	0	3
I:Rough-O:-:Enx	C2	0	0	0	1	0	1
I:Rough-O:B:1 2 -:B:2	B	1	0	0	0	0	1
I:Rough-O:Eh:1 5 -:Eh:5	B	0	0	2	0	0	2
I:Rough-O:Gm:--:Gm:-	D	1	0	0	0	0	1
I:Rough-O:R:1 2	B	1	0	0	0	0	1
I:Rough-O:B:-	C1	0	1	0	0	0	1
I:Rough-O:E,H:-	C2	0	0	1	0	0	1
I:Rough-O:G,M:-	D	0	0	0	1	0	1
I:Rough-O:i:z6	C2	7	0	0	0	0	7
I:Rough-O:r:-	B	1	0	0	0	0	1
I:Rough-O:r:1,2	B	1	0	0	0	0	1
Iiia:23:-:-	G2	2	0	0	0	0	2
Iiia:23:G,z51:-	G2	1	0	0	0	0	1
Indiana	B	0	0	0	1	7	8
Infantis	C1	17	0	0	0	0	17
Kentucky	C2	**667**	8	6	3	0	**684**
Livingstone	C1	24	1	2	0	0	27
London	E1	0	1	0	3	0	4
Mbandaka	C1	25	0	0	0	0	25
Montevideo	C1	2	1	0	0	0	3
Muenster	E1	2	0	3	0	0	5
Newport	C2	0	0	10	0	0	10
Ohio	C1	0	**44**	0	0	0	44
Oranienburg	C1	1	0	0	0	0	1
Orion	E2	5	0	**44**	0	0	49
Saintpaul	B	1	0	4	0	0	5
Schwarzengrund	B	4	0	7	0	0	11
Senftenberg	E4	18	4	**94**	**28**	1	**145**
Tennessee	C1	1	0	0	3	0	4
Thompson	C1	4	2	0	1	0	7
Typhimurium	B	24	**22**	3	0	**26**	**75**
Uganda	E1	0	0	5	2	4	11
Worthington	G2	0	0	0	0	1	1
**Total**		**1014**	**105**	**305**	**209**	**45**	**1678**

Within a column, the serovars with the highest frequency are shown in bold.

**Table 4:** Frequency of *Salmonella enterica* ser. Enteritidis phage types (PTs) isolated from fluff samples submitted through the Ontario Hatchery and Supply Flock Policy between 2009 and 2018 (*n* = 233 samples).

Phage Type	Count
PT 2	1
PT 5b	1
PT 8	28
PT 9b	37
PT 13	11
PT 13a	11
PT 19	2
PT 22	3
PT 23	1
PT 51	4
Atypical	92
Not Typed ^1^	39
Untypable	3
**Total**	**233**

^1^ Samples that were submitted after September 2017 and were not phage typed.

**Table 5:** Statistically significant (*p* ≤ 0.05) temporal clusters ^1^ of *Salmonella enterica* serovars isolated from fluff samples submitted through the Ontario Hatchery and Supply Flock Policy between 2009 and 2018, by poultry commodity.

Poultry Commodity	Serovar ^2^	First Cluster ^3^	Second Cluster ^3^	Third Cluster ^3^	Fourth Cluster ^3^	Fifth Cluster ^3^	Sixth Cluster ^3^
Broiler hatcheries	Enteritidis (76)	2009/1–2009/4 (17) ^4^	2010/10–2011/2 (12)	2017/10–2018/2 (11)			
	Heidelberg (88)	2010/8–2012/10 (55)	2015/8–2015/8 (7)	2009/7–2009/11 (8)			
	Kentucky (668)	2009/1–2013/10 (483)	2015/4–2017/6 (123)	2013/12–2014/4 (19)			
	Livingstone (24)	2017/12–2018/1 (6)					
	Mbandaka (25)	2015/8–2018/11 (24)					
	Senftenberg (18)	2015/1–2015/5 (5)					
	Typhimurium (24)	2009/6–2009/7 (9)					
Layer hatcheries	Ohio (44)	2016/11–2017/9 (35)	2010/9–2010/9 (3)	2018/1–2018/1 (3)	2013/4–2013/4 (2)		
	Typhimurium (22)	2015/10–2015/10 (4)	2014/1–2014/3 (6)				
Turkey hatcheries	Heidelberg (99)	2012/8–2013/2 (50)	2009/5–2010/5 (21)	2012/7–2015/11 (26)			
	Newport (10)	2011/3–2011/5 (8)					
	Orion (44)	2010/9–2012/2 (43)					
	Senftenberg (94)	2014/10–2014/10 (11)	2015/7–2015/7 (8)	2009/1–2012/6 (52)	2016/11–2016/12 (7)	2017/8-2017/8 (4)	2015/4-2015/4 (3)
Waterfowl hatcheries	Enteritidis (155)	2015/1–2018/12 (83)	2012/1–2013/12 (54)	2011/1–2014/12 (16)			
	Senftenberg (28)	2009/1–2009/12 (14)	2012/1–2012/12 (6)				
Game-bird hatcheries	Typhimurium (26)	2014/5–2018/12 (21)					

^1^ Iterative temporal scan performed using SaTScan v9 with a scanning window size of 50% of the study period (Kulldorff, 2018). ^2^ Total number of isolates is given in parentheses. ^3^ Number of isolates identified in each cluster is given in parentheses. ^4^ Dates are given in a year/month format.

**Table 6:** Comparison of the Akaike information criterion (AIC) statistics calculated for a logistic regression model without random intercepts, and logistic regression models with random intercepts for hatchery and date of sample collection (*n* = 25,303 fluff samples from 5022 visits from 42 hatcheries).

Type of Model and Fixed Effects	AIC
**Without random effects**	
Poultry commodity, season, year	12,059.04
**Two random effects (hatchery, date of sample collection)**	
Poultry commodity, season, year	10,037.71
Poultry commodity*season, year	10,019.88
Poultry commodity*year, season	9970.63
Poultry commodity, season*year	10,038.67
Poultry commodity*year, season*poultry commodity	**9938.12**

The best-fitting model (lowest AIC score) is shown in bold. An asterisk between fixed effects indicates an interaction term between the two variables.

**Table 7:** Multivariable logistic regression model of factors (poultry commodity, year, season) associated with the presence of *Salmonella enterica* isolated from fluff samples submitted through the Ontario Hatchery and Supply Flock Policy between 2009 and 2018, with random effects for hatchery and date of sample collection (*n* = 25,303 observations from 5022 visits from 42 hatcheries).

Variable	Category	OR ^1^	*p*-Value	95% Confidence Interval	
Year	**2009**	**Referent**			
	2010	1.64	0.059	0.98	2.78
	2011	1.51	0.117	0.90	2.53
	2012	1.08	0.754	0.64	1.83
	2013	1.002	0.995	0.58	1.74
	2014	0.28	**<0.001**	0.15	0.51
	2015	0.58	**0.048**	0.33	1.00
	2016	0.40	**0.001**	0.23	0.69
	2017	0.39	**0.001**	0.23	0.67
	2018	0.24	**<0.001**	0.13	0.42
Poultry Commodity	**Broiler hatcheries**	**Referent**			
	Layer hatcheries	0.07	**0.013**	0.01	0.56
	Game-bird hatcheries	0.02	**0.017**	0.00	0.48
	Turkey hatcheries	0.15	0.144	0.01	1.94
	Waterfowl hatcheries	0.41	0.436	0.04	3.87
Season	**Winter**	**Referent**			
	Spring	1.14	0.468	0.80	1.64
	Summer	1.006	0.976	0.70	1.45
	Fall	1.004	0.982	0.69	1.46
Year*Poultry Commodity	2009*Broiler hatcheries	**Referent**			
	2010*Layer hatcheries	0.86	0.843	0.19	3.83
	2011*Layer hatcheries	0.39	0.257	0.08	2.00
	2012*Layer hatcheries	0.23	0.155	0.03	1.75
	2013*Layer hatcheries	1.83	0.381	0.47	7.12
	2014*Layer hatcheries	4.27	**0.041**	1.06	17.14
	2015*Layer hatcheries	1.84	0.390	0.46	7.40
	2016*Layer hatcheries	2.43	0.206	0.61	9.65
	2017*Layer hatcheries	0.74	0.741	0.13	4.33
	2018*Layer hatcheries	0.91	0.935	0.09	8.77
	2010*Game-bird hatcheries	3.30	0.412	0.19	57.10
	2011*Game-bird hatcheries	2.19	0.590	0.13	37.53
	2012*Game-bird hatcheries	4.51	0.285	0.29	71.06
	2013*Game-bird hatcheries	4.54	0.304	0.25	81.38
	2014*Game-bird hatcheries	56.25	**0.005**	3.49	907.40
	2015*Game-bird hatcheries	29.76	**0.017**	1.85	479.90
	2016*Game-bird hatcheries	56.75	**0.005**	3.40	946.59
	2017*Game-bird hatcheries	220.54	**<0.001**	13.65	3563.81
	2018*Game-bird hatcheries	44.80	**0.013**	2.23	899.27
	2010*Turkey hatcheries	0.68	0.415	0.27	1.71
	2011*Turkey hatcheries	0.68	0.405	0.27	1.69
	2012*Turkey hatcheries	0.62	0.313	0.25	1.57
	2013*Turkey hatcheries	0.39	0.064	0.14	1.06
	2014*Turkey hatcheries	1.95	0.294	0.56	6.83
	2015*Turkey hatcheries	1.81	0.320	0.56	5.86
	2016*Turkey hatcheries	0.43	0.228	0.11	1.71
	2017*Turkey hatcheries	0.31	0.119	0.07	1.35
	2018*Turkey hatcheries	0.50	0.367	0.11	2.24
	2010*Waterfowl hatcheries	0.17	**0.019**	0.04	0.74
	2011*Waterfowl hatcheries	0.27	0.093	0.06	1.24
	2012*Waterfowl hatcheries	4.10	**0.039**	1.08	15.6
	2013*Waterfowl hatcheries	5.86	**0.010**	1.52	22.54
	2014*Waterfowl hatcheries	4.61	**0.032**	1.14	18.65
	2015*Waterfowl hatcheries	7.32	**0.005**	1.84	29.02
	2016*Waterfowl hatcheries	4.95	**0.023**	1.25	19.59
	2017*Waterfowl hatcheries	11.42	**0.001**	2.80	46.56
	2018*Waterfowl hatcheries	9.22	**0.002**	2.25	37.73
Season*Poultry Commodity	Winter*Broiler hatcheries	**Referent**			
	Spring*Layer hatcheries	0.86	0.746	0.34	2.16
	Spring*Game-bird hatcheries	0.93	0.930	0.19	4.66
	Spring*Turkey hatcheries	0.62	0.189	0.31	1.26
	Spring*Waterfowl hatcheries	2.12	0.097	0.87	5.15
	Summer*Layer hatcheries	0.60	0.301	0.23	1.58
	Summer*Game-bird hatcheries	3.97	0.075	0.87	18.12
	Summer*Turkey hatcheries	3.26	**<0.001**	1.71	6.23
	Summer*Waterfowl hatcheries	3.42	**0.010**	1.34	8.72
	Fall*Layer hatcheries	0.53	0.215	0.20	1.44
	Fall*Game-bird hatcheries	2.60	0.229	0.55	12.37
	Fall*Turkey hatcheries	2.09	**0.030**	1.07	4.09
	Fall*Waterfowl hatcheries	2.45	0.054	0.98	6.10
σ^2^ (Hatchery)		5.04		2.75	9.23
σ^2^ (Visit)		1.94		1.60	2.36

Significant variables (*p* ≤ 0.05) are shown in bold. An asterisk between fixed effects indicates an interaction term between the two variables. ^1^ Odds ratio.

## Data Availability

All relevant data are included in the manuscript.

## Supplementary Materials

The following is available online at https://www.mdpi.com/article/10.3390/pathogens11010009/s1, Figure S1: Predicted probability of *Salmonella enterica* isolated from fluff samples submitted through the Ontario Hatchery and Supply Flock Policy between 2009 and 2018, by poultry commodity. (**a**): broiler hatcheries; (**b**): layer hatcheries; (**c**): turkey hatcheries; (**d**): waterfowl hatcheries; (**e**): game-bird hatcheries. The y-axis scale may differ between graphs so that details can be observed.

## References

[1] Government of Canada (2018). National Enteric Surveillance Program Annual Summary 2017.

[2] World Health Organization *Salmonella* (non-typhoidal). https://www.who.int/news-room/fact-sheets/detail/salmonella-(non-typhoidal).

[3] Thomas M.K., Murray R., Flockhart L., Pintar K., Fazil A., Nesbitt A., Marshall B., Tataryn J., Pollari F. (2015). Estimates of Foodborne Illness-Related Hospitalizations and Deaths in Canada for 30 Specified Pathogens and Unspecified Agents. Foodborne Pathog. Dis..

[4] Varga C., John P., Cooke M., Majowicz S.E. (2020). Spatial and Space-Time Clustering and Demographic Characteristics of Human Nontyphoidal *Salmonella* Infections with Major Serotypes in Toronto, Canada. PLoS ONE.

[5] Emond-Rheault J.G., Jeukens J., Freschi L., Kukavica-Ibrulj I., Boyle B., Dupont M.J., Colavecchio A., Barrere V., Cadieux B., Arya G. (2017). A Syst-OMICS Approach to Ensuring Food Safety and Reducing the Economic Burden of Salmonellosis. Front. Microbiol..

[6] Government of Canada (2018). Foodnet Canada Annual Report 2017.

[7] Varga C., Pearl D.L., McEwen S.A., Sargeant J.M., Pollari F., Guerin M.T. (2013). Incidence, Distribution, Seasonality, and Demographic Risk Factors of *Salmonella* Enteritidis Human Infections in Ontario, Canada, 2007–2009. BMC Infect. Dis..

[8] Middleton D., Savage R., Tighe M.K., Vrbova L., Walton R., Whitfield Y., Varga C., Lee B., Rosella L., Dhar B. (2014). Risk Factors for Sporadic Domestically Acquired *Salmonella* serovar Enteritidis Infections: A Case-Control Study in Ontario, Canada, 2011. Epidemiol. Infect..

[9] Snyder T.R., Boktor S.W., M’ikanatha N.M. (2019). Salmonellosis Outbreaks by Food Vehicle, Serotype, Season, and Geographical Location, United States, 1998 To 2015. J. Food Prot..

[10] Marus J.R., Magee M.J., Manikonda K., Nichols M.C. (2019). Outbreaks of *Salmonella enterica* Infections Linked to Animal Contact: Demographic and Outbreak Characteristics and Comparison to Foodborne Outbreaks—United States, 2009–2014. Zoonoses Public Health.

[11] Anderson T.C., Marsden-Haug N., Morris J.F., Culpepper W., Bessette N., Adams J.K., Bidol S., Meyer S., Schmitz J., Erdman M.M. (2017). Multistate Outbreak of Human *Salmonella* Typhimurium Infections Linked to Pet Hedgehogs—United States, 2011–2013. Zoonoses Public Health.

[12] Varga C., Middleton D., Walton R., Savage R., Tighe M.K., Allen V., Ahmed R., Rosella L. (2012). Evaluating Risk Factors for Endemic Human *Salmonella* Enteritidis Infections with Different Phage Types in Ontario, Canada using Multinomial Logistic Regression and a Case-Case Study Approach. BMC Public Health.

[13] Collineau L., Phillips C., Chapman B., Agunos A., Carson C., Fazil A., Reid-Smith R.J., Smith B.A. (2020). A Within-Flock Model of *Salmonella* Heidelberg Transmission in Broiler Chickens. Prev. Vet. Med..

[14] Liljebjelke K.A., Hofacre C.L., Liu T., White D.G., Agers S., Young S., Maurer J.J. (2005). Vertical and Horizontal Transmission of *Salmonella* within Integrated Broiler Production System. Foodborne Pathog. Dis..

[15] Braden C. (2006). *Salmonella enterica* serotype Enteritidis and Eggs: A National Epidemic in the United States. Clin. Infect. Dis..

[16] Davies R.H., Wales A.D. (2014). Developments in *Salmonella* Control in Eggs. Adv. Microb. Food Saf..

[17] Jensen A.N., Dalsgaard A., Stockmarr A., Nielsen E.M., Baggesen D.L. (2006). Survival and Transmission of *Salmonella enterica* serovar Typhimurium in an Outdoor Organic Pig Farming Environment. Appl. Environ. Microbiol..

[18] Sivaramalingam T., Pearl D.L., McEwen S.A., Ojkic D., Guerin M.T. (2013). A temporal Study of *Salmonella* Serovars from Fluff Samples from Poultry Breeder Hatcheries in Ontario between 1998 and 2008. Can. J. Vet. Res..

[19] Guerin M.T., Martin S.W., Darlington G.A., Rajic A. (2005). A Temporal Study of *Salmonella* Serovars in Animals in Alberta between 1990 and 2001. Can. J. Vet. Res..

[20] Government of United States of America National Enteric Disease Surveillance: Salmonella Annual Report, 2012: Center for Emerging and Infectious Diseases, USA. https://www.cdc.gov/ncezid/dfwed/pdfs/salmonella-annual-report-2012-508c.pdf.

[21] Government of United States of America National Enteric Disease Surveillance: Salmonella Annual Report, 2013: Center for Emerging and Infectious Diseases, USA. https://www.cdc.gov/ncezid/dfwed/pdfs/covis-annual-report-2013-508c.pdf.

[22] Government of United States of America National Enteric Disease Surveillance: Salmonella Annual Report, 2011: Center for Emerging and Infectious Diseases, USA. http://www.cdc.gov/nationalsurveillance/PDFs/NationalSalmSurveillOverview_508.pdf.

[23] Government of United States of America National Enteric Disease Surveillance: Salmonella Annual Report, 2009: Center for Emerging and Infectious Diseases, USA. https://www.cdc.gov/ncezid/dfwed/pdfs/salmonella-annual-summary-2009-508c.pdf.

[24] Van Heeswyk B. (2021). Personal communication.

[25] Varga C., Pearl D.L., McEwen S.A., Sargeant J.M., Pollari F., Guerin M.T. (2015). Spatial-Temporal Epidemiology of Human *Salmonella* Enteritidis Infections with Major Phage Types (PTs 1, 4, 5b, 8, 13, and 13a) in Ontario, Canada, 2008–2009. BMC Public Health.

[26] Government of Canada (2018). National Enteric Surveillance Program Annual Summary 2018.

[27] Murray C.E., Varga C., Ouckama R., Guerin M.T. Temporal Study of *Salmonella enterica* Serovars Isolated from Environmental Samples from Ontario Poultry Breeder Flocks between 2009 and 2018.

[28] Sivaramalingam T., McEwen S.A., Pearl D.L., Ojkic D., Guerin M.T. (2013). A Temporal Study of *Salmonella* Serovars from Environmental Samples from Poultry Breeder Flocks in Ontario between 1998 and 2008. Can. J. Vet. Res..

[29] Government of United States of America (2015). Serotypes Profile of *Salmonella* Isolates from Meat and Poultry Products: United States Department of Agriculture, Food Safety and Inspection Service. https://www.fsis.usda.gov/sites/default/files/media_file/2020-10/Salmonella-Serotype-Annual-2014.pdf.

[30] Caffrey N., Agunos A., Gow S., Liljebjelke K., Mainali C., Checkley S.L. (2021). *Salmonella* spp. Prevalence and Antimicrobial Resistance in Broiler Chicken and Turkey Flocks in Canada from 2013 to 2018. Zoonoses Public Health.

[31] Sander J., Hudson C.R., Dufour-Zavala L., Waltman W.D., Lobsinger C., Thayer S.G., Otalora R., Maurer J.J. (2001). Dynamics of *Salmonella* Contamination in a Commercial Quail Operation. Avian Dis..

[32] Pathogen Safety Data Sheets: Infectious Substances—*Salmonella enterica* spp.. https://www.canada.ca/en/public-health/services/laboratory-biosafety-biosecurity/pathogen-safety-data-sheets-risk-assessment/salmonella-enterica.html..

[33] Forshell L.P., Wierup M. (2006). *Salmonella* Contamination: A Significant Challenge to the Global Marketing of Animal Food Products. Rev.Sci. Tech. Off. Int. Epiz..

[34] Government of Canada Licensed Hatcheries in Canada, 2021: Canadian Food Inspection Agency, Canada. https://inspection.canada.ca/animal-health/terrestrial-animals/diseases/licensed-hatcheries-in-canada/eng/1464108675209/1464109620337.

[35] Kulldorff M. (2018). Information Management Services I. SaTScanTM v 9.6: Software for the Spatial, Temporal, and Space-Time Scan Statistics. www.satscan.org.

[36] Kulldorff M. (1997). A Spatial Scan Statistic. Commun. Stat. Theory Methods.

