# Supplementary material for: Temporal Study of Salmonella enterica Serovars Isolated from Fluff Samples from Ontario Poultry Hatcheries between 2009 and 2018
Source: Pathogens. 2021 Dec 22;11(1):9. doi: 10.3390/pathogens11010009 (PMC8781519; doi:10.3390/pathogens11010009)
Supplement: Supplementary file 1 [file pathogens-11-00009-s001.zip › pathogens-1465089-supplementary.pdf]

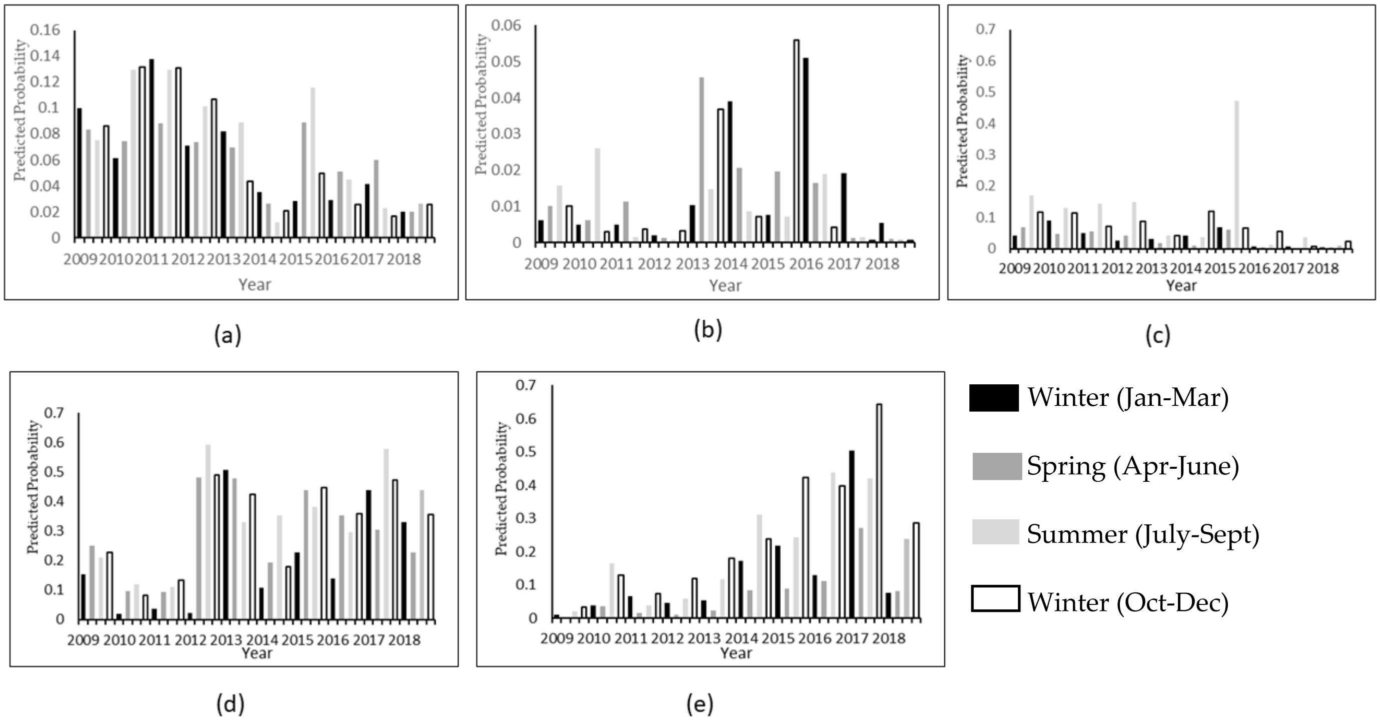

Figure S1: Predicted probability of Salmonella enterica isolated from fluff samples submitted through the Ontario Hatchery and Supply Flock Policy between 2009 and 2018, by poultry commodity (a=broiler hatcheries, b=layer hatcheries, c=turkey hatcheries, d=waterfowl hatcheries, and e=game bird hatcheries). The y-axis scale may differ between graphs so that details can be observed.
